# Are Diet Preferences Associated to Skulls Shape Diversification in Xenodontine Snakes?

**DOI:** 10.1371/journal.pone.0148375

**Published:** 2016-02-17

**Authors:** Julia Klaczko, Emma Sherratt, Eleonore Z. F. Setz

**Affiliations:** 1 Departamento de Biologia Animal, Instituto de Biologia, Universidade Estadual de Campinas, UNICAMP, Campinas, SP, Brazil; 2 School of Environmental and Rural Science, University of New England, Armidale, New South Wales, Australia; Monash University, AUSTRALIA

## Abstract

Snakes are a highly successful group of vertebrates, within great diversity in habitat, diet, and morphology. The unique adaptations for the snake skull for ingesting large prey in more primitive macrostomatan snakes have been well documented. However, subsequent diversification in snake cranial shape in relation to dietary specializations has rarely been studied (e.g. piscivory in natricine snakes). Here we examine a large clade of snakes with a broad spectrum of diet preferences to test if diet preferences are correlated to shape variation in snake skulls. Specifically, we studied the Xenodontinae snakes, a speciose clade of South American snakes, which show a broad range of diets including invertebrates, amphibians, snakes, lizards, and small mammals. We characterized the skull morphology of 19 species of xenodontine snakes using geometric morphometric techniques, and used phylogenetic comparative methods to test the association between diet and skull morphology. Using phylogenetic partial least squares analysis (PPLS) we show that skull morphology is highly associated with diet preferences in xenodontine snakes.

## Introduction

The origin and diversification of morphology are topics of great interest with the field of evolutionary biology, and the adaptation of organismal form to ecological conditions has been attributed as a primary driving force of morphological diversification [1]. Classic support for the hypothesis of adaptation by natural selection is evolutionary convergence. Evolutionary convergence occurs when similar phenotypes evolve in phylogenetically independent taxa as a response to similar ecological conditions [2, 3]. Due to the importance of the skull, and its direct link to an animal’s fitness, it is presumed that skull morphology is under considerably strong selection pressure [4]. Among the many ecological functions of the skull, feeding is one of the most essential; and presumably, diet can influence the skull [5, 6]. Many studies within vertebrates have corroborated this idea, showing strong correlations between diet and skull morphology [6, 7, 8]. Stayton [9] studied lizard skulls shape evolution across 17 families, using geometric morphometric tools. He showed morphological convergent evolution among lizards with similar diets. However, these patterns were secondary over the more general phylogenetic pattern of lizard skull diversity.

Snakes are a highly successful group of vertebrates, with large diversity in habits, environments, diet, and morphology [10]. They comprise more than 3000 species, widely distributed across temperate and tropical regions, with diverse dietary preferences [11]. Those historical shifts probably resulted in adaptive radiations that contributed to the high diversity of species observed today [12, 13].

Snakes, particularly macrostomatans, can feed on very large prey, much larger that the size of their heads. Snakes with macrostoman condition possess highly kinetic skulls, which allow for the transport of the entire prey through their oral cavities using ratcheting motions of the lower jaws [11, 14, 12, 15]. Genomic phylogenies suggest that this capacity have evolved twice, in tropidophiids, basal alethinophidians, and the families Bolyeriidae, Tropidophiidae, Boidae, and Pythonidae, plus the "advanced snakes" that include Acrochordidae and Colubroidea grouped as Caenophidia [16]. Several studies have suggested association between specific types of diet and the morphology of snakes [17, 18]. However, to our knowledge, no study has used geometric morphometric tools and phylogenetic comparative methods to explicitly test the association between diet and skull morphology in snakes.

**Fig 1:**
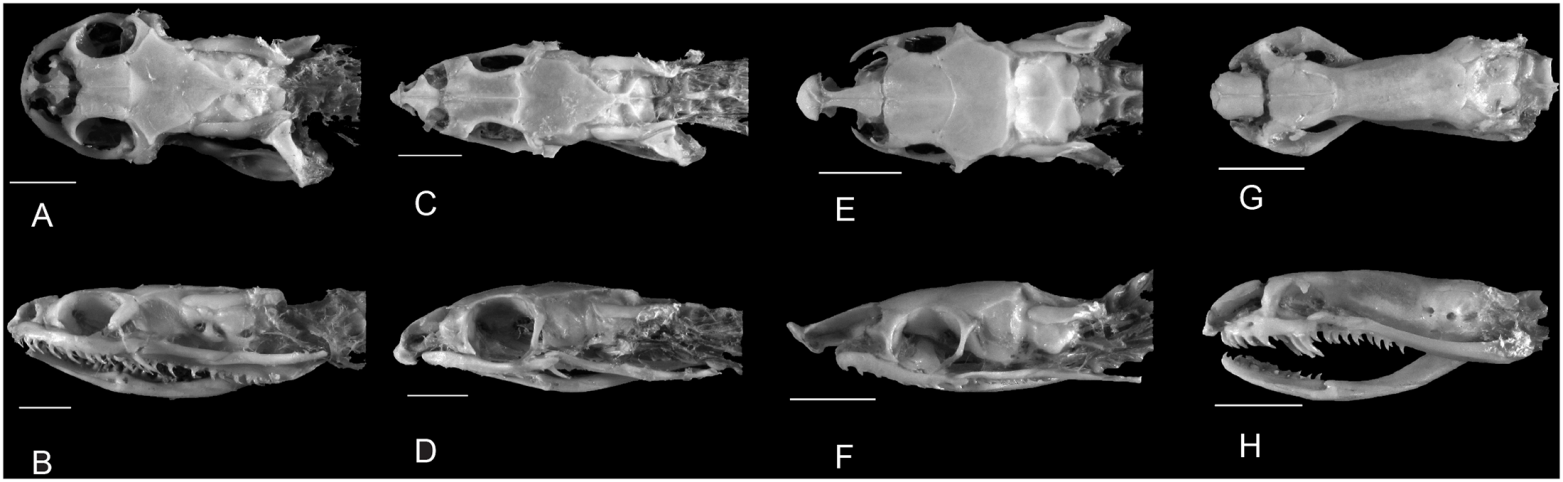
Example of morphological variation in Xenodontinae snake skulls. Dorsal view (A) and lateral view (B) of *Helicops angulatus* UFMT 7818, an eater of fish, lizards, and anurans; Dorsal view (C) and lateral view (D) of *Philodryas aestivus* IBSP 6771, an eater of mammals and anurans; Dorsal view (E) and lateral view (F) of *Phimophis guerini* IBSP 66406, an eater of lizards and mammals; Dorsal view (G) and lateral view (H) of *Phalotris mertensi* ZUEC 486, an eater of elongate vertebrates (caecilians and amphisbaenians).

Xenodontines are a speciose clade of South American snakes that include 49 genera and approximately 330 species [19]. Molecular phylogenetic hypotheses corroborate the monophyly of the group [20], and are well known for their great diversity in morphology and ecological features (Fig 1, [21]). In particular, xenodontine snakes show a broad range of diets including invertebrates, amphibians, lizards, snakes and small mammals [22]; and so are a good representative of the diet preference diversity found among modern macrostomatan snakes. The high level of morphological and ecological diversity makes xenodontine snakes an ideal group to study mechanisms that promote lineage diversification and evolutionary radiations in macrostomatan snakes [21].

Here we describe the variation in skull shape of 19 species of xenodontine snakes using geometric morphometric techniques [23; 24]. Using dietary preferences compiled from the literature with phylogenetic comparative methods we addressed the following question: is the variation of skull shape in xenodontine snakes influenced only by the speciation process, or has the diet fostered the diversification of skull shape?

## Material and Methods

### Data

We examined skull morphology in 19 species of South American xenodontine snakes (range 1–5 specimens per species, mean of 4), representing the diversity of diet preferences present in xenodontine snakes. The analyzed specimens were from the following museums: Museu Nacional, Rio de Janeiro, Brazil (MNRJ), Instituto Butantan, São Paulo, Brazil (IBSP), Universidade Federal do Mato Grosso, Cuiabá, Brazil (UFMT), Museu de Zoologia “Prof. Adão José Cardoso”, Unicamp, Campinas, São Paulo, Brazil (ZUEC). The complete list of the analyzed specimens can be found in the Supporting Information, Table A in S1 File. The skulls were dissected from museum specimens and skeletonized by hand. We obtained digital images of the skulls in dorsal and lateral views, using a Canon PowerShot S5 SI digital camera. Images were standardized for skull position, camera lens plane position, and distance between camera lens and skull.

**Fig 2:**
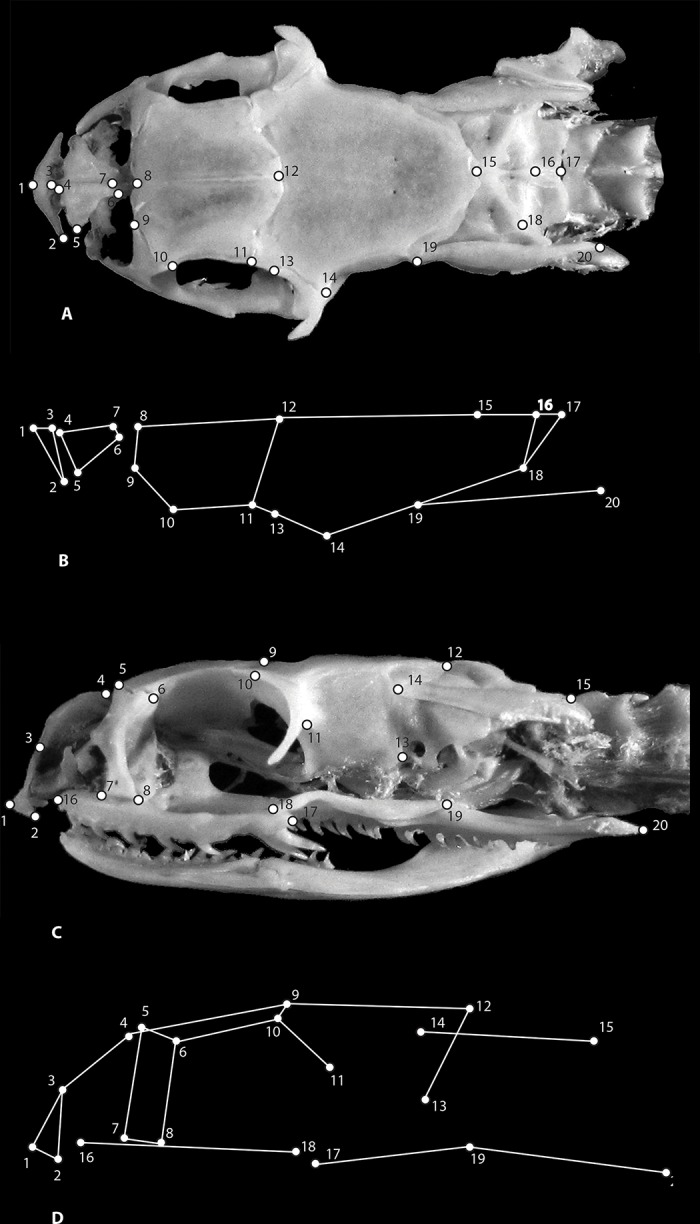
Cranial landmarks. Cranial landmarks recorded from South American Xenodontinae snakes. Dorsal view (A), anatomical wire frame of dorsal view (B), lateral view (C), and anatomical wire frame of lateral view (D).

One of us (JK) digitized a set of 20 landmarks in each view (dorsal and lateral) of the skull (Fig 2, for landmarks definitions see list on Table B in S1 File) using tpsDig 2.12 [25]. The landmarks were digitized only on one side of the skull, since our focus was not on the asymmetric component of shape [26]. The landmark data were aligned using Generalized Procrustes Analysis [27; 23]. Then the average shape for each species was calculated and used for subsequent analyses.

Principal Component Analysis was performed on the average shape for all species Procrustes shape coordinates to visualize the variation among species. We obtained 18 principal axes (PCs) for each view that describes the shape variation. The differences in shape described by each principal axis (PC) were summarized using thin-plate spline deformation grids [28; 29]. The analyses were performed using Geomorph package v.2.1 [29; 30] for R software v.3.1.1 [31], and tpsRelw 1.46 software [25].

Diet preferences were obtained from literature, and are presented as the proportion of each item on the diet (Table 1).

**Table 1:** Proportion of prey items recorded for xenodontinae snakes; an = anuran amphibians; bi = birds; ca = caecilids or amphisbenids; eg = lizard eggs; ew = earthworms; fi = fishes; li = lizards; ma = mammals; mo = mollusks; sn = snakes.

	mo	ew	fi	an	ca	li	sn	bi	eg	ma	Other	Reference
*Apostolepis assimilis*					1.00							[32]
*Boiruna maculata*						0.1	0.58	0.16		0.13	0.03	[33]
*Elapomorphus quinquelineatus*					0.94				0.06			[34, 45]
*Erythrolamprus aesculapii*				0.04			0.96					[35]
*Gomesophis brasiliensis*		1.00										[36]
*Helicops angulatus*			0.81	0.14		0.05						[37]
*Hydrodynastes gigas*			0.42	0.54						0.04		[38]
*Lystrophis dorbignyi*						0.94			0.06			[39]
*Oxyrhopus rhombifer*						0.49	0.02			0.49		[33]
*Phalotris mertensi*					1.00							[32]
*Philodryas aestiva*						0.5				0.5		[32]
*Phimophis guerini*						0.92				0.08		[33]
*Psomophis joberti*						0.5				0.5		[38]
*Siphlophis pulcher*						0.83	0.1		0.07			[32]
*Taeniophallus affinis*				0.9	0.05	0.05						[37]
*Tomodon dorsatus*	1.00											[40]
*Tropidodryas striaticeps*				0.04		0.11	0.01	0.18		0.66		[41]
*Uromacer catesbyi*				0.31		0.69						[42]
*Xenoxybelis argentus*				0.52		0.47						[43]

### Phylogeny and Comparative Approach

The phylogenetic relationships between the xenodontine species were reconstructed using *Dipsas indica* as the outgroup. We used sequences of mitochondrial genes 12S and 16S rDNA, and nuclear gene Oocyte maturation factor-like (c-mos). All sequences were obtained from GenBank, and aligned using ClustalW [44] (List of used accession numbers–Table C in S1 File). jModelTest 0.1 [45] was employed to determine the most appropriate model of sequence evolution for each analyzed gene, estimated under the Bayesian Information Criterion (BIC). The best fit model for the genes 12S and 16S was TPM3uf + G, and for the gene c-mos was TPM2uf. The combined molecular data set was analyzed under the Bayesian Inference method, implementing the selected optimal sequence evolution model for each gene. The phylogenetic analysis was performed using MrBayes v3.1.2 [46]. Two runs were performed, with default heating for each of the four chains and sampling every 100 generations for 20,000,000 generations. The convergence was verified using Tracer v1.4 [47]; and the first 5000 topologies (25% of the sampled topologies) were discarded as burn-in.

The phylogenetic signal skull shape (the Procrustes shape coordinates) was evaluated using the multivariate K statistic (see [48]) in the R package Geomorph v.2.1. The K statistic can indicate little or no phylogenetic signal (K << 1), or phylogenetic signal (K ≈ 1), or greater than the expectation under a Brownian motion random-walk model of evolution (K >> 1). To test if the data contain a signal of phylogeny (i.e. K > 0), we randomly permuted the order of species on the tree 1000 times and recalculated K for each permutation. We then compared the observed K value to this null distribution to assess significance.

To visualize the evolutionary history of the analyzed of skull shape, the consensus phylogenetic tree was projected onto the shape space. We used the squared-change parsimony method implemented in MorphoJ [49], which is equivalent to maximum likelihood methods when branch lengths are present.

To characterize the evolutionary patterns of covariation between skull shape variation and diet preferences we used phylogenetic two-block partial least squares analysis (PPLS; [50]). Partial least squares is a statistical procedure that quantifies the degree of covariation between sets of variables, based on a singular value decomposition of the overall trait covariance matrix [51]. The phylogenetic PLS uses instead the evolutionary covariance matrix and assumes the expected lack of independence among samples as a result of phylogenetic relationship [51]. Since our consensus phylogenetic tree shows some polytomies, the PPLS analysis was conducted on 200 resolved trees randomly selected from MrBayes output. The significance of the model was assessed employing a permutation test. The PPLS was performed in the Geomorph package v.2.1.

## Results

The principal components (PC) analysis of the skull shapes reveals that most of the shape variation is contained in few dimensions; in the dorsal view the first four PCs explained 91.5%, while in the lateral view the first four PCs explained 88.3% of the total shape variation.

**Fig 3:**
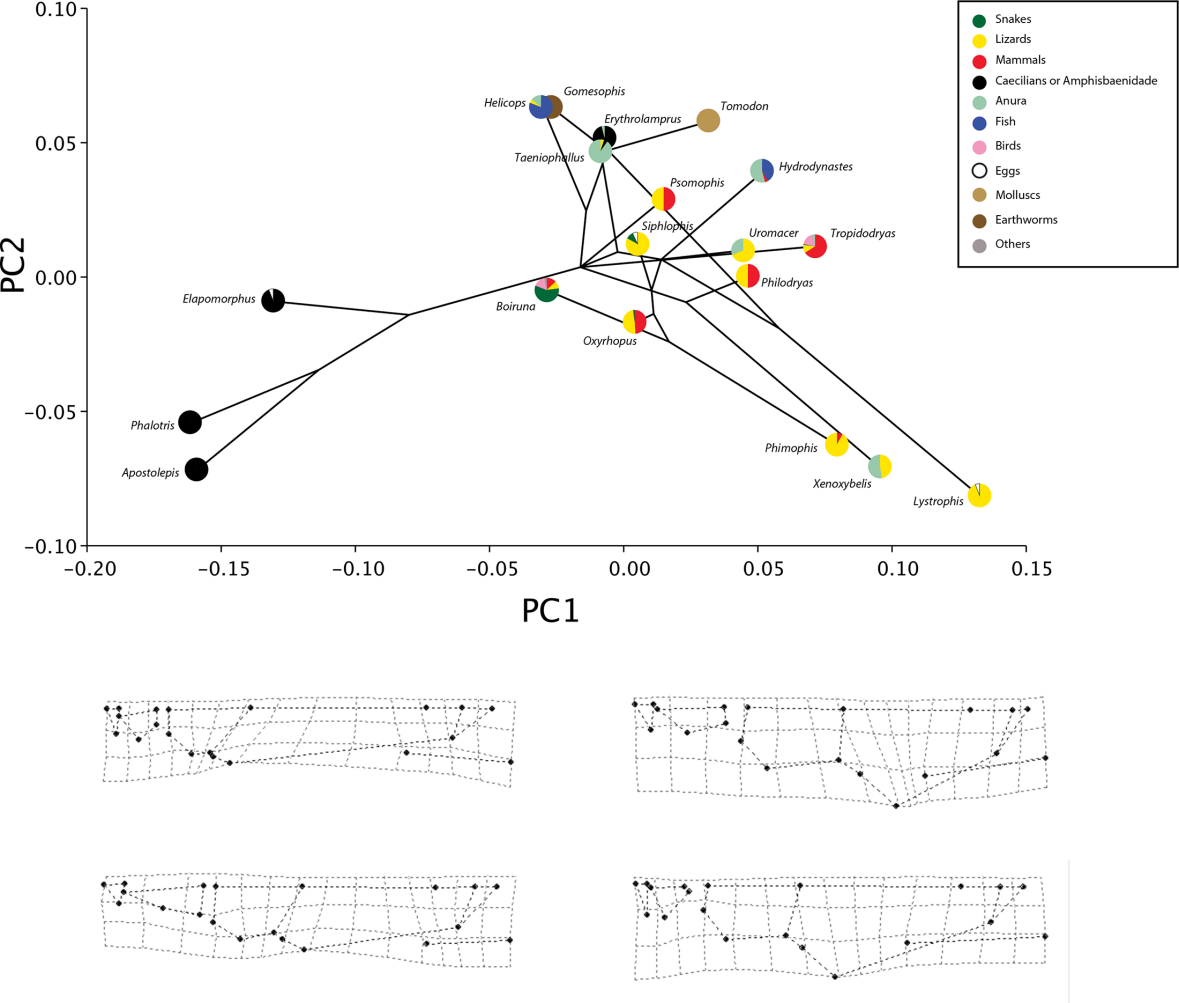
The phylomorphospace of dorsal cranial shape. (A) Projection of the phylogenetic tree into the dorsal view PC morphospace. (B) Estimated changes in dorsal view shape are shown as deformations from the mean shape along the first and second principal components.

**Fig 4:**
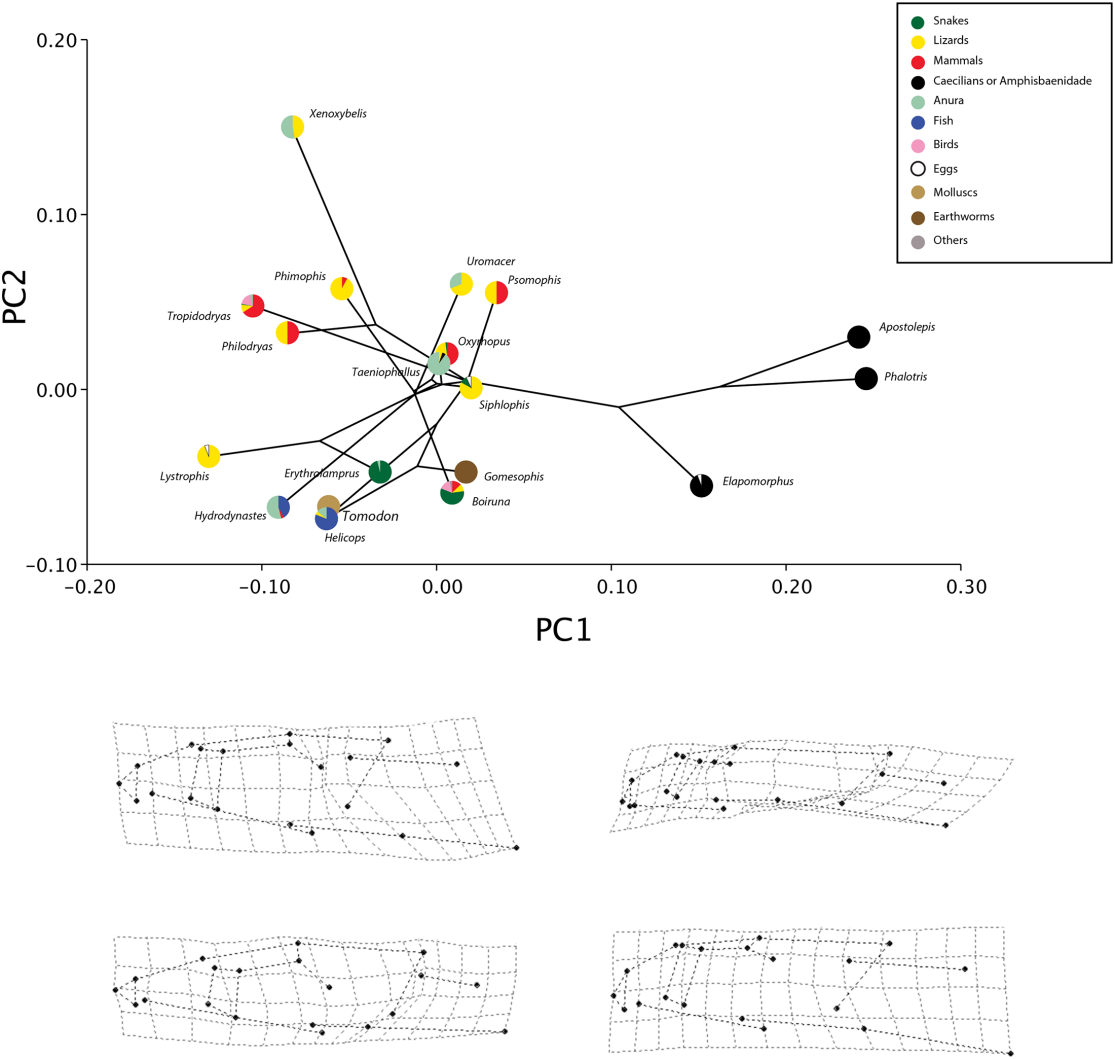
The phylomorphospace of lateral cranial shape. (A) Projection of the phylogenetic tree into the lateral view PC morphospace. (B) Estimated changes in lateral view shape are shown as deformations from the mean shape along the first and second principal components.

In the dorsal view, PC1 explained 56.4% of the variation, and the species with negative values show an increase of skull length, especially of the parietal bone, and a relative decrease in orbit size; while positive values indicate a wider and shorter skull (Fig 3). On the other hand, in the lateral view the PC1 explained 54.4% of the variation; positive values of the PC1, on the right side of the graph, show a more robust and shortened skull, especially the maxilla bone, while species with negative values of PC1, show a more elongated jaw (Fig 4).

The molecular phylogeny based on Bayesian Inference analysis was generally consistent with previous phylogenetic hypotheses, we found support for the monophyly of all the xenodontine tribes [19; 20], (Figure A in S1 File). Our phylogeny showed the clade *Erythrolamprus* and *Lystrophis* associated to the clade that contain *Siphlophis*, *Oxyrhopus*, *Boiruna* and *Phimophis* while Vidal et al. [19] showed *Erythrolamprus* and *Lystrophis* as a more inclusive clade sister group of the clade containing *Helicops*, *Tomodon*, *Gomesophis*, *Siphlophis*, *Oxyrhopus*, *Boiruna* and *Phimophis*. On the other hand, Pyron et al. [20] did not included *Elapomorphus* in their analysis. They showed *Psomophis* as a sister group of the clade formed by *Uromacer*, *Erythrolamprus*, and *Lystrophis*.

According to the phylogenetic signal metric K, there is significant phylogenetic signal in cranial shape. For the dorsal view, the mean K values from 200 trees is 0.95 (*P*_mean_ = 0.005) and similarly for lateral view, K mean is 0.98 (*P*_mean_ = 0.003) (see the histogram of K and *P* values in S1 Fig). Despite the strong phylogenetic signal results, projecting the consensus phylogenetic tree into the shape space for each view of the skull demonstrates a striking pattern (Figs 3 and 4). Some sister-taxa are widely separated in shape space, indicating they have very different morphologies. Species from Elapomorphini tribe are separated from all other species along PC1 and are more similar to each other in morphology, and share the same diet.

Xenodontine skull shape is highly correlated with diet. PPLS of dorsal skull shape on diet across the 200 trees resulted in a mean value of the degree of covariance accessed by permutation tests of 0.87 (*P*_mean_ = 0.003), and for lateral skull shape a mean value of the degree of covariance of 0.84 (*P*_mean_ = 0.01) (see the histogram of the values in S2 Fig).

In order to determine whether the species from Elapomorphini tribe (*Apostolepis assimilis*, *Elapomorphus quinquelineatus*, and *Phalotris mertensi*) were driving the association between diet preferences and skull morphology, we repeated the PPLS analyses of both skull views after excluding these taxa from the consensus tree. The dorsal skull shape resulted in a correlation of 0.80 (*P* = 0.007), and the lateral skull shape a correlation of 0.81 (*P* = 0.02), thus these results indicate that this clade has little influence on the overall results.

## Discussion

Our results show that skull shape morphology in xenodontine snakes is strongly associated with diet [52]. Many studies have suggested association between snake skull morphology and diet in snakes. Maxillary teeth have been associated to different types of diet pronounced posterior ridges located on the posterior maxillary teeth are associated with slug predators [53] long, sharper teeth and elongated mandible bones are associated with piscivory [53], and enlarged anterior teeth and arched maxilla are thought to help *Lycodon aulicus capucinus* to ingest hard-bodied skinks [54]. Natricine piscivorous snakes were broadly studied, and the results show that fish-eating snakes have relatively longer skulls than frog-eating snakes, which tended to have broader skull components [55, 56, 57]. Studies analyzing the diversity of diet preferences usually focus on a broad scale analysis, for example, Vincent et al. [16] analyzed 12 monophyletic clades across macrostomatan snakes and found that head width is significantly related to the mean of the consumed prey mass, suggesting that skull in snakes is adapted to prey size (see also [14; 11]). Only few studies analyzed the evolution of skull morphology and diet at finer scales, such as the diversity of diet within a family or a genus [33].

Our study is the first to both describe snake skull morphology using geometric morphometric tools, and to correlate it to a broad spectrum of diet preferences using phylogenetic comparative methods in a highly diverse snake subfamily. However, our results alone are not able to state that diet drives the diversification of the skull morphology [52]. Our results highlighted a high correlation between diet and skull shape in xenodontine snakes; however we fail to define causality. To do so, it would be necessary to future studies of skull functionality and behavior directly associated to feeding.

The phylogenetic partial least squares analyses showed high correspondence of skull shape and diet that can be interpreted as an indicative of evolutionary convergence. Morphological convergence is an excellent opportunity to study adaptation at the macroevolutionary scale, since it offers multiple independent tests of the morphological response to similar functional demands [58]. However, as Revell *et al*. [2] pointed, morphological similarities among taxa could be a result of several causal processes, including evolutionary convergence, and evolutionary parallelism. Evolutionary convergence occurs when lineages with different ancestral morphologies evolve to the same phenotype through different directions, while in evolutionary parallelism lineages with the same ancestral morphology evolve in the same direction toward the same phenotype. In other words, parallelism is a special case of convergent evolution where independent evolutionary lineages evolve the same trait using the same genes and/or developmental pathways, while convergence occurs when lineages evolve similar traits using different developmental pathways. Species that evolve under parallel evolution are likely to share similar patterns of genetic covariation, and as a result, convergent evolution of a trait may occur not because selection favored that trait, but because it favored the same correlated trait in each species. As a consequence, natural selection could be favoring a correlated trait, and not the focal one, and any conclusion should carefully draw from evolutionary convergence and natural selection [59]. A more comprehensive sampling of taxa, combined with a robust and well resolved phylogeny of xenodontine snakes, is required to distinguish between these alternative hypotheses.

Vincent et al. [14], based on the analysis of skull size variation across several snakes species, showed that the increase in head width in snakes is followed by changes in jaw length and lower jaw out-lever length, suggesting that the snakes skulls were highly integrated. Skull parts are integrated because each part tightly associated to the other. Morphological integration can be a result of developmental, functional, genetic, and/or evolutionary interaction and constraints [60]. On the other hand, the skull is also modular, since morphological integration is not uniform throughout the entire skull, and it can be divided in modules that are strongly integrated internally, but are relatively independent of other modules [61]. Evolutionary integration and modularity can be investigated by examining how evolutionary changes in multiple parts are coordinated across a set of related species, and using comparative methods to take into account the phylogenetic structure of the variation [60]. Future studies are needed to test the modularity and integration among snake skull parts to better picture the forces influencing skull shape evolution.

## Supporting Information

S1 Fig Phylogenetic Signal of skull shape across 200 phylogenetic trees. (A) Lateral cranial shape *Phylogenetic signal* values and (B) *P* values. (C) Dorsal cranial shape *Phylogenetic signal* values and (D) *P* values.
(TIF)

S2 Fig Phylogenetic Partial Least Squares of skull shape on diet across 200 phylogenetic trees. (A) Lateral cranial shape correlations values and (B) *P* values. (C) Dorsal cranial shape correlations values and (D) *P* values.
(TIF)

S1 File List of analyzed material (Table A). Cranial landmarks definitions recorded from South American Xenodontinae snakes (Table B). Genbank access numbers (Table C). South American Xenodontinae phylogeny. Bayesian inference phylogeny (mtDNA 12S and 16S, and c-mos) of South American Xenodontinae. Numbers on the branches represent the posterior probabilities (Figure A).
(DOCX)
