# Supplementary figures and images for: Are Diet Preferences Associated to Skulls Shape Diversification in Xenodontine Snakes?
Source: PLoS One. 2016 Feb 17;11(2):e0148375. doi: 10.1371/journal.pone.0148375 (PMC4757418; doi:10.1371/journal.pone.0148375)

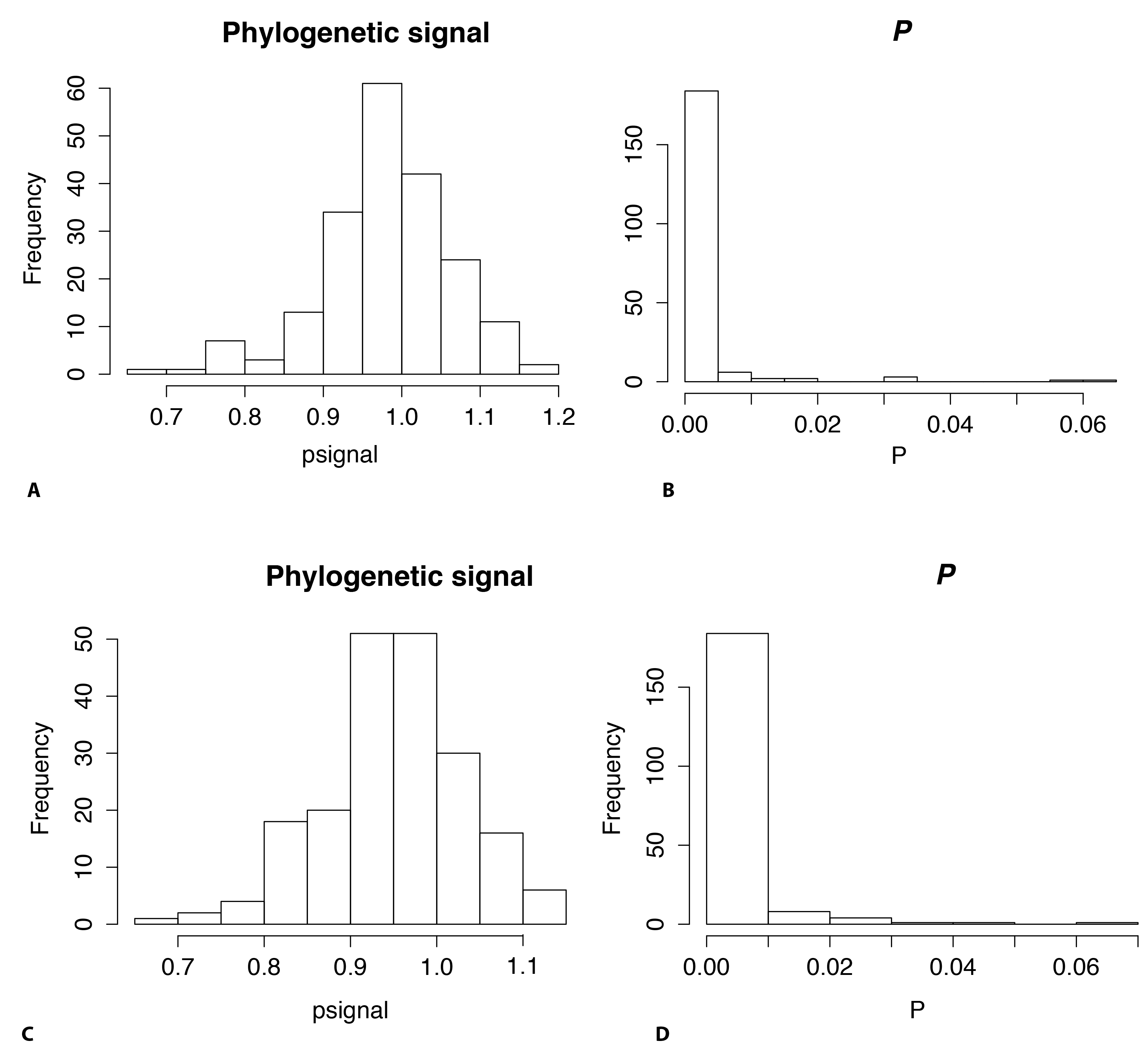

Supplement: S1 Fig — (A) Lateral cranial shape Phylogenetic signal values and (B) P values. (C) Dorsal cranial shape Phylogenetic signal values and (D) P values. (TIF) [file pone.0148375.s001.tif]

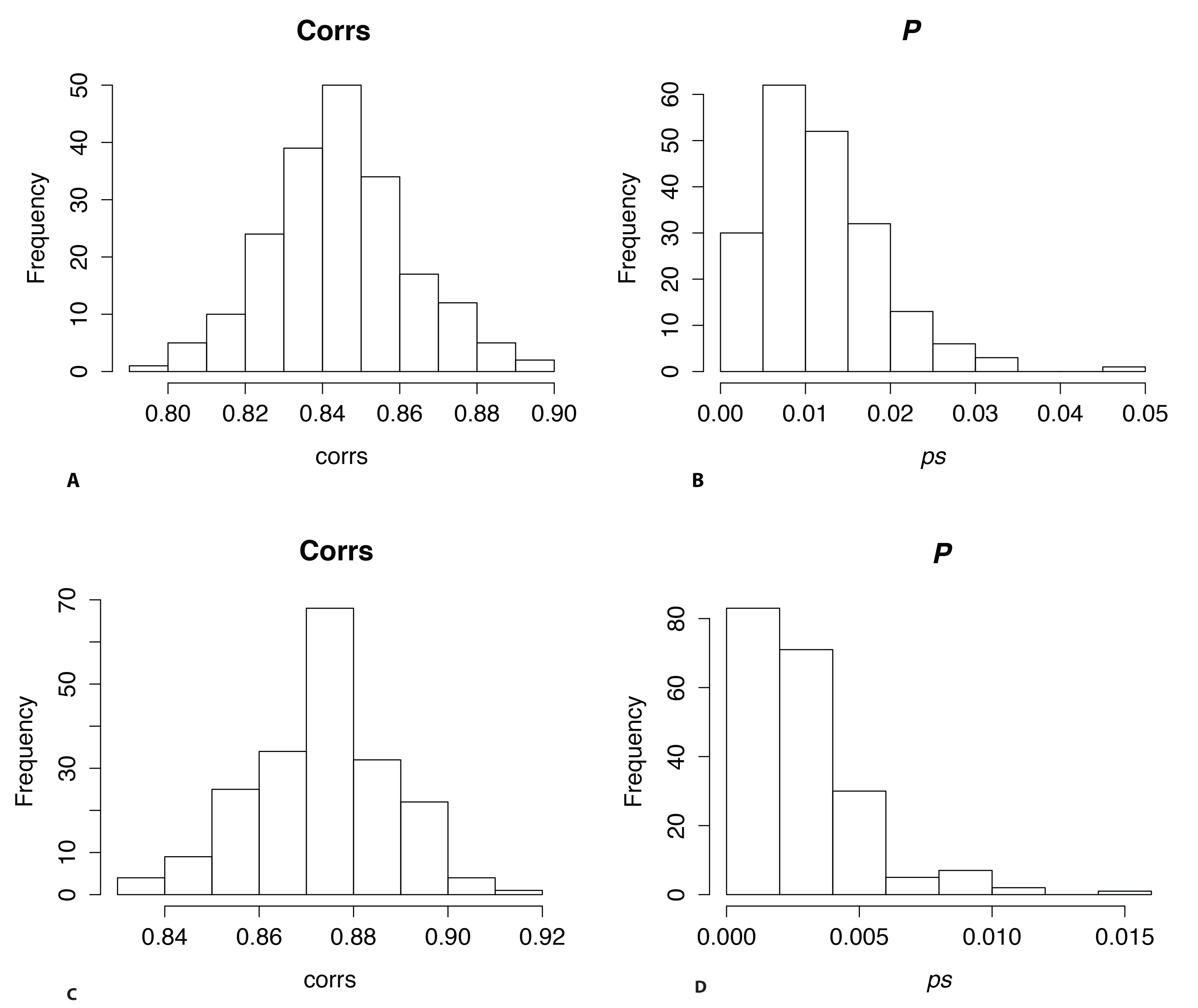

Supplement: S2 Fig — (A) Lateral cranial shape correlations values and (B) P values. (C) Dorsal cranial shape correlations values and (D) P values. (TIF) [file pone.0148375.s002.tif]
