# Supplementary material for: Are Diet Preferences Associated to Skulls Shape Diversification in Xenodontine Snakes?
Source: PLoS One. 2016 Feb 17;11(2):e0148375. doi: 10.1371/journal.pone.0148375 (PMC4757418; doi:10.1371/journal.pone.0148375)
Supplement: S1 File — Bayesian inference phylogeny (mtDNA 12S and 16S, and c-mos) of South American Xenodontinae. Numbers on the branches represent the posterior probabilities (Figure A). (DOCX) [file pone.0148375.s003.docx]

**Table A.** **List of analyzed material.** The analyzed specimens were loan from the following museums: Museu Nacional, Rio de Janeiro, Brazil (MNRJ), Instituto Butantan, São Paulo, Brazil (IBSP), Universidade Federal do Mato Grosso, Cuiabá, Brazil (UFMT), Museu de Zoologia “Prof. Adão José Cardoso”, Unicamp, Campinas, São Paulo, Brazil (ZUEC).

| **Genus** | **Species** | **Voucher** | |
| --- | --- | --- | --- |
| *Apostolepis* | *assimilis* | IBSP | 3998 |
| *Apostolepis* | *assimilis* | IBSP | 8449 |
| *Apostolepis* | *assimilis* | ZUEC | 1267 |
| *Apostolepis* | *assimilis* | ZUEC | 2356 |
| *Apostolepis* | *assimilis* | ZUEC | 2754 |
| *Boiruna* | *maculata* | UFMT | 813 |
| *Boiruna* | *maculata* | UFMT | 1934 |
| *Boiruna* | *maculata* | ZUEC | 946 |
| *Boiruna* | *maculata* | ZUEC | 1084 |
| *Boiruna* | *maculata* | ZUEC | 1085 |
| *Elapomorphus* | *quinquilineatus* | IBSP | 1290 |
| *Elapomorphus* | *quinquilineatus* | IBSP | 10057 |
| *Elapomorphus* | *quinquilineatus* | IBSP | 32130 |
| *Elapomorphus* | *quinquilineatus* | MNRJ | 19488 |
| *Erythrolamprus* | *aesculapii* | UFMT | 286 |
| *Erythrolamprus* | *aesculapii* | UFMT | 483 |
| *Erythrolamprus* | *aesculapii* | ZUEC | 54 |
| *Erythrolamprus* | *aesculapii* | ZUEC | 105 |
| *Erythrolamprus* | *aesculapii* | ZUEC | 530 |
| *Erythrolamprus* | *aesculapii* | ZUEC | 562 |
| *Gomesophis* | *brasiliensis* | IBSP | 1704 |
| *Gomesophis* | *brasiliensis* | IBSP | 16954 |
| *Gomesophis* | *brasiliensis* | IBSP | 17107 |
| *Helicops* | *angulatus* | UFMT | 2811 |
| *Helicops* | *angulatus* | UFMT | 7816 |
| *Helicops* | *angulatus* | ZUEC | 1015 |
| *Helicops* | *angulatus* | ZUEC | 1590 |
| *Helicops* | *angulatus* | ZUEC | 1942 |
| *Hydrodynastes* | *gigas* | IBSP | 36999 |
| *Hydrodynastes* | *gigas* | IBSP | 37132 |
| *Hydrodynastes* | *gigas* | UFMT | 19 |
| *Hydrodynastes* | *gigas* | UFMT | 5836 |
| *Lystrophis* | *dorbignyi* | IBSP | 23196 |
| *Lystrophis* | *dorbignyi* | IBSP | 23197 |
| *Lystrophis* | *dorbignyi* | MNRJ | 549 |
| *Oxyrhopus* | *rhombifer* | IBSP | s/n |
| *Oxyrhopus* | *rhombifer* | UFMT | 933 |
| *Oxyrhopus* | *rhombifer* | UFMT | 5154 |
| *Oxyrhopus* | *rhombifer* | ZUEC | 1169 |
| *Oxyrhopus* | *rhombifer* | ZUEC | 2498 |
| *Oxyrhopus* | *rhombifer* | ZUEC | 3088 |
| *Oxyrhopus* | *rhombifer* | ZUEC | 3097 |
| *Phalotris* | *mertensi* | ZUEC | 486 |
| *Phalotris* | *mertensi* | ZUEC | 1579 |
| *Phalotris* | *mertensi* | ZUEC | 1688 |
| *Phalotris* | *mertensi* | ZUEC | 2011 |
| *Phalotris* | *mertensii* | IBSP | 9955 |
| *Philodryas* | *aestivus* | IBSP | 743 |
| *Philodryas* | *aestivus* | IBSP | 765 |
| *Philodryas* | *aestivus* | IBSP | 6771 |
| *Philodryas* | *aestivus* | IBSP | 7617 |
| *Phimophis* | *guerini* | IBSP | 66404 |
| *Phimophis* | *guerini* | IBSP | 66406 |
| *Phimophis* | *guerini* | MNRJ | 14205 |
| *Phimophis* | *guerini* | UFMT | 600 |
| *Phimophis* | *guerini* | UFMT | 603 |
| *Phimophis* | *guerini* | UFU | 327 |
| *Psomophis* | *joberti* | IBSP | 65466 |
| *Psomophis* | *joberti* | IBSP | 65551 |
| *Psomophis* | *joberti* | MNRJ | 3347 |
| *Psomophis* | *joberti* | UFMT | 4081 |
| *Psomophis* | *joberti* | UFMT | 4082 |
| *Siphlophis* | *pulcher* | IBSP | 42219 |
| *Siphlophis* | *pulcher* | IBSP | 68396 |
| *Siphlophis* | *pulcher* | ZUEC | 1636 |
| *Siphlophis* | *pulcher* | ZUEC | 2226 |
| *Taeniophalus* | *affinis* | IBSP | 19749 |
| *Taeniophalus* | *affinis* | IBSP | 51561 |
| *Taeniophalus* | *affinis* | MNRJ | 17468 |
| *Tomodon* | *dorsatus* | IBSP | 16850 |
| *Tomodon* | *dorsatus* | IBSP | 16987 |
| *Tomodon* | *dorsatus* | IBSP | 17122 |
| *Tomodon* | *dorsatus* | IBSP | 17547 |
| *Tomodon* | *dorsatus* | IBSP | 17891 |
| *Tomodon* | *dorsatus* | IBSP | 18425 |
| *Tomodon* | *dorsatus* | IBSP | 20793 |
| *Tomodon* | *dorsatus* | IBSP | 20795 |
| *Tropidodryas* | *striaticeps* | IBSP | 9633 |
| *Tropidodryas* | *striaticeps* | ZUEC | 412 |
| *Tropidodryas* | *striaticeps* | ZUEC | 856 |
| *Tropidodryas* | *striaticeps* | ZUEC | 1008 |
| *Tropidodryas* | *striaticeps* | ZUEC | 2103 |
| *Uromacer* | *catesbyi* | IBSP | 242 |
| *Xenoxybelis* | *argentus* | UFMT | 7293 |
| *Xenoxybelis* | *argentus* | UFMT | 7982 |

**Table B.** Cranial landmarks definitions recorded from South American Xenodontinae snakes. See Figure 2.

| **Dorsal view landmarks definition (Fig. 2 A and B)** | |
| --- | --- |
| 1 | midline point on the premaxillar at the inferior tip of the bony septum; |
| 2 | tip of the palatine process on the premaxillar; |
| 3 | tip of the nasal process on the premaxillar; |
| 4 | anterior-midline of nasal; |
| 5 | lateral tip of nasal; |
| 6 | tip of the frontal process on the nasal; |
| 7 | posterior-midline of nasal; |
| 8 | anterior-midline of frontal; |
| 9 | anterior frontal-prefrontal suture; |
| 10 | posterior frontal-prefrontal suture; |
| 11 | frontal-parietal lateral suture; |
| 12 | frontal-parietal midline suture; |
| 13 | anterior postorbital-parietal suture; |
| 14 | posterior postorbital-parietal suture; |
| 15 | parietal-supraoccipital suture, midline; |
| 16 | supraoccipital-exoccipital suture, midline; |
| 17 | supraoccipital-exoccipital suture, lateral; |
| 18 | Exoccipital posterior tip, midline; |
| 19 | anteriormost tip of supratemporal; |
| 20 | posteriormost tip of supratemporal |
|  |  |
| **Lateral view landmarks definition (Fig. 2 C and D)** | |
| 1 | midline point on the premaxillar at the inferior tip of the bony septum; |
| 2 | tip of the palatine process on the premaxillar; |
| 3 | tip of the nasal process on the premaxillar; |
| 4 | nasal-frontal suture; |
| 5 | anterior frontal-prefrontal suture; |
| 6 | posterior frontal-prefrontal suture; |
| 7 | anterior prefrontal-maxilla suture; |
| 8 | posterior prefrontal-maxilla suture; |
| 9 | posterior frontal-prefrontal suture; |
| 10 | anterior postorbital-parietal suture; |
| 11 | posterior postorbital-parietal suture; |
| 12 | parietal-supraoxipital suture, midline |
| 13 | anteriormost tip of supratemporal; |
| 14 | posteriormost tip of supratemporal; |
| 15 | anteriormost tip of maxilla; |
| 16 | end of maxilla; |
| 17 | anteriormost tip of ectopterigoid; |
| 18 | end of ectopterigoid; |
| 19 | anteriormost tip of pterigoid; |
| 20 | end of pterigoid. |

**Table C. Genbank access numbers.**

| **Species** | **12S** | **16S** | **C-mos** |
| --- | --- | --- | --- |
| *Apostolepis assimilis* | *GQ457781* | *GQ457724* | *GQ457843* |
| *Boiruna maculata* | *GQ457785* | *–* | *GQ457847* |
| *Elapomorphus quinquelineatus* | *GQ457794* | *GQ457736* | *GQ457855* |
| *Erythrolamprus aesculapii* | *GQ457795* | *GQ457736* | *GQ457856* |
| *Gomesophis brasiliensis* | *GQ457796* | *GQ457737* | *–* |
| *Helicops angulatus* | *GQ457797* | *GQ457738* | *GQ457857* |
| *Hydrodynastes gigas* | *GQ457803* | *GQ457743* | *GQ457863* |
| *Lystrophis dorbignyi* | *GQ457812* | *GQ457752* | *GQ457872* |
| *Oxyrrhopus rhombifer* | *GQ457816* | *GQ457755* | *GQ457876* |
| *Phalotris mertensi* | *GQ457818* | *GQ457757* | *GQ457878* |
| *Philodryas aestivus* | *GQ457819* | *GQ457758* | *GQ457879* |
| *Phimophis guerini* | *GQ457822* | *GQ457761* | *–* |
| *Psomophis joberti* | *GQ457829* | *GQ457768* | *GQ457889* |
| *Siphlophis pulcher* | *GQ457834* | *GQ457773* | *GQ457894* |
| *Taeniophallus affinis* | *GQ457792* | *GQ457733* | *GQ457853* |
| *Tomodon dorsatus* | *GQ457838* | *GQ457777* | *GQ457897* |
| *Tropidodryas striaticeps* | *GQ457839* | *GQ457778* | *GQ457853* |
| *Uromacer catesbyi* | *AF158454* | *AF158523* | *–* |
| *Xenoxybelis argenteus* | *GQ457842* | *GQ457780* | *GQ457899* |

**Fig A. South American Xenodontinae phylogeny**. Bayesian inference phylogeny (mtDNA 12S and 16S, and c-mos) of South American Xenodontinae, numbers on the branches represent the posterior probabilities.
